# Clasped Plates

**Published:** 1869-07

**Authors:** 


### ARTICLE VIII.

### *Clasped Plates.*

Professor Smith at a recent meeting of the "Pennsylvania Odontographic Society," thought the subject under consideration "The Relative Merits of Clasps and Atmospheric Pressure for Retaining Partial Plates," to be one of great practical importance, affecting as it does the appearance and comfort of a very considerable number of patients, while often taxing the skill and jeopardizing the reputation of the dentist. A matter of so much moment demands careful study, and requires that in forming conclusions, we be assisted by sound judgement and practical experience. Very opposite opinions have been expressed this evening as to the efficiency and utility of the different modes of applying partial dentures—some claiming that in no case can an artificial piece be retained in the mouth by other means than atmospheric pressure, without positive injury to the natural teeth; others giving as the result of their trials that from one to three of the oral teeth inserted without the aid of clasps, cannot be retained in the mouth during the process of mastication. Speaking from his own observation, he could not acquiesce in the views presented by either side. He would go neither to the one extreme nor the other, but stand upon middle ground; this he believed most emphatically a tenable position, sustained by facts and the teaching of everyday practice. While in most cases his preference would be for suction plates, yet he unhesitatingly discarded the theory of of necesitated and positive injury from every form of clasped denture.

English dentists, for more than half a century, have used partial plates clasped, almost exclusively ; their testimony is not such as to lead to utter condemnation, but rather to a continuance of the usage. Much injury has unquestionably been done to the natural teeth by clasps ; but he believed that in almost every instance it could be directly traced to the neglect or want of cleanliness on the part of the patient; to the injudicious selection of natural organs to which to apply them ; to the improper form of the support, or the want of adaptation. If our American students were as thoroughly schooled in the manipulations pertaing to dental mechanism as the English are, he beleived far less mischief would be observed from clasped plates. There is too great a tendency to confound as synonymous in these days of the reign of cheap materials, the ability to adjust a set of suction teeth on a base of rubber, and mechanical dentistry. It is here, in a want of knowledge of the principles of mechanical dentistry, that we find the source of evil from clasped plates ; just in proportion as we understand these principles, and by nice manipulations are able to put them in practice, will we diminish the injury to the natural teeth from the use of bands. This style, when well adjusted, will need a clasp only to steady it ; its *main* support being from accuracy of adaptation to the parts upon which it rests. Often, very often, plates are formed and clasps adjusted in such a manner as to compel them to do *all* the work of sustaining the piece. Where such is the case, detriment to the natural organs must be the result.

Classes of teeth entirely unsuited for such a purpose are not infrequently selected as supports for partial cases. He had often seen clasps about the cuspid teeth, and in one or two instances about central incisors.

That a clasped plate necessitated the absorption of the processes about the natural teeth more than a suction plate would do, he believed to be without foundation in fact. In regard to a clasp about a natural tooth interfering with its normal condition, as a string embracing muscular tissue interfers with its functions, he thought we had no evidence to justify us in concluding. The damage which is done to a tooth he conceived to be purely external, and when the plate is properly formed and the clasps nicely adjusted, there is little danger from this source. A marked want of attention to the cleanliness of the piece, assisting the mechanical action of the clasp, may be, and doubtless is, a prolific source of harm.

While he believed the clasping of partial cases, in the present condition of dental prosthesis, to be decidedly *good* practice, he nevertheless discarded the views that suction plates cannot be made to answer where the teeth had no antagonists. He felt no hesitation in inserting from one to six oral teeth on suction plates, when such a course seems most in harmony with the requirements of the case; had a number of such in his own practice, and had seen them from the hands of various gentlemen in the profession.

An objection urged against suction plates for partial cases, was the difficulty of obtaining a perfect impression in wax. He recommended plaster; where there is a liability of displacing the wax in withdrawing from the mouth, it is the material to meet the demands; it does not draw but breaks, and in such a manner as to preclude the possibility of getting it into any position but the correct one when adjusting the broken pieces. The cup may be detached from the plaster in the mouth, then cut, if hot, so as to break in a manner to best facilitate removal. One of the most valuable properties of plaster as an impression material is its quality of resisting displacement, when set, without breaking. With plaster, as perfect an impression of the mouth can be obtained for a partial as for an entire case.

In regard to chambers, he was satisfied that the very best form of suction plates were those without them; as commonly made they are too deep. A shallow cavity is far more efficient than a deep one, as the part is only put upon the stretch, while the deep is soon filled, often with an indurated mass, which renders it worse than useless. Professor Smith called attention to the spring plates patented by E. B. Goodall, of New Hampshire, and explained the manner of constructing them. Objection being raised to this method be-of the patent, he confessed his inability to understand why cause a professional man, simply because he is such, should be debarred from protecting an invention by legally obtaining a patent, while the mechanic is applauded for such a course.

He considered mechanical dentistry, so called, by far the most perplexing department of dentistry, requiring for its intelligent practice an extended range of experience and information. He hailed with open arms any discovery or invention, patent or otherwise, that would assist in securing more certain and satisfactory results than have yet been reached in this branch.

---

